# Antibacterial and Antibiofilm Activity of Green-Synthesized Zinc Oxide Nanoparticles Against Multidrug-Resistant *Escherichia coli* Isolated from Retail Fish

**DOI:** 10.3390/molecules30040768

**Published:** 2025-02-07

**Authors:** Mohamed Tharwat Elabbasy, Rasha M. El Bayomi, Esraa A. Abdelkarim, Abd El-Salam E. Hafez, Mohamed S. Othman, Mohamed E. Ghoniem, Mai A. Samak, Muteb H. Alshammari, Fahad Awwadh Almarshadi, Tamer Elsamahy, Mohamed A. Hussein

**Affiliations:** 1 Department of Pathology, College of Medicine, University of Ha’il, Ha’il 55476, Saudi Arabia; tharwat330@gmail.com (M.T.E.); dr.maiamin@yahoo.com (M.A.S.); 2 Food Hygiene, Safety, and Technology Department, Faculty of Veterinary Medicine, Zagazig University, Zagazig 44511, Egypt; rmazab@vet.zu.edu.eg (R.M.E.B.); elged2010@yahoo.com (M.A.H.); 3 Department of Biochemistry, College of Medicine, University of Ha’il, Ha’il 55476, Saudi Arabia; mo.abdelkarim@uoh.edu.sa; 4 Department of Internal Medicine, College of Medicine, University of Ha’il, Ha’il 2240, Saudi Arabia; teto2006.km@gmail.com; 5 Department of Health Informatics, College of Public Health and Health Informatics, University of Ha’il, Ha’il 55476, Saudi Arabia; muteb.h.alshammari@gmail.com; 6 Department of Public Health, College of Public Health and Health Informatics, University of Ha’il, Ha’il 55476, Saudi Arabia; fa.alotaibi@uoh.edu.sa; 7 Independent Researcher, Zhenjiang 212013, China

**Keywords:** fish pathogens, zinc oxide nanoparticles, multidrug resistance, virulence factor, food safety, nanoparticle bioactivity

## Abstract

Multidrug-resistant (MDR) *Escherichia coli* is a major foodborne pathogen posing a critical threat to public health, particularly through the contamination of animal products. The increasing prevalence and virulence of MDR *E. coli* strains underscore the urgent need for alternative antimicrobial strategies. This study aimed to synthesize and characterize zinc oxide nanoparticles (ZnO-NPs) using *Stevia rebaudiana* as a sustainable capping and reducing agent, aligning with green chemistry principles. Of the 120 fish samples, 74.2% (89/120) were positive for *E. coli* contamination. Among the identified *E. coli* strains, 77.8% (119/153) were classified as MDR. Resistance profiling revealed 22 distinct patterns, and seven highly resistant and virulent strains were selected for further analyses. The eco-friendly auto-combustion synthesis of ZnO-NPs produced nanoparticles with semi-spherical to hexagonal shapes and an average size ranging from 12 to 25 nm. Scanning Electron Microscope–Energy Dispersive X-ray analysis (SEM-EDS) confirms that ZnO-NPs primarily consist of zinc (37.5%) and oxygen (19.9%), with carbon (42.6%) indicating the green synthesis process. ZnO-NPs demonstrated potent, dose-dependent antibacterial and antibiofilm activity against the selected MDR *E. coli* strains. Additionally, mechanistic studies revealed that ZnO-NPs disrupt bacterial cell membranes, alter cellular morphology, and interfere with DNA integrity. These findings highlight the potential of eco-friendly ZnO-NPs as a promising nanomaterial for enhancing food safety and addressing the growing challenge of MDR foodborne bacteria.

## 1. Introduction

Nile tilapia (*Oreochromis niloticus*), Atlantic mackerel (*Scomber scombrus*), and European sardine (*Sardina pilchardus*) are vital sources of nutrition, offering essential proteins, amino acids, vitamins, and minerals crucial for human health [1]. Additionally, fish are important sources of micronutrients (e.g., calcium and iron), which contribute to bone health and metabolic processes [2]. However, bacterial infection in aquaculture presents a growing concern, threatening the quality of fish products and public health. Bacterial pathogens can cause fish diseases, leading to increased mortality rates and reduced marketability due to stringent health and safety standards [3]. Moreover, consuming contaminated fish poses significant health risks to humans, including the risk of foodborne illnesses. This underscores the need for rigorous biosecurity measures in aquaculture to safeguard both fish health and consumer safety.

Multidrug-resistant (MDR) *Escherichia coli* has emerged as a significant threat, associated with high morbidity, mortality, and substantial economic losses [4]. The rise and spread of MDR bacterial strains have exacerbated the challenges of managing bacterial infections in several fish species [5,6,7]. Genetic mutations, horizontal gene transfer, and the improper use of antibiotics drive this resistance. These threats arise from the limited treatment options available and the increased risk of treatment failures in humans and animals. For example, *E. coli* was identified as the prevalent pathogenic bacterium in retail fish in the United States [7] and India [8], accounting for 67% and 65% of tested samples.

Nanotechnology, a multidisciplinary field, has gained significant global attention because of the unique properties of nanoparticles (NPs), including their different shapes, small size, and high surface area. These properties make NPs suitable for several medical applications, pharmaceuticals, cosmetics, and the food industry [9]. Zinc oxide nanoparticles (ZnO-NPs) are particularly interesting due to their exceptional electrical, photostability, and semiconductor properties, and their chemical stability [10]. ZnO-NPs have significant potential in nanomedicine, gene delivery, drug delivery, biological sensing, and labeling technologies [11]. Conventional synthesis methods for ZnO-NPs, such as sol–gel, hydrothermal, and mechanochemical techniques, are often time-consuming, costly, and require high temperatures and specific precursors, which generate significant chemical waste [12]. In contrast, NP green synthesis employs renewable organic extracts from yeast, bacteria, and plants, eliminating the need for harmful chemicals and contributing to waste reduction. By leveraging agricultural waste rich in secondary metabolites, this approach provides a sustainable alternative for nanoparticle production, aligning with ecological synthesis principles [13,14,15]. For example, Naiel et al. [16] synthesized ZnO-NPs using Sea Lavender extract, producing NPs (41 nm) that showed potent antioxidant activity and potential antimicrobial activity towards *E. coli* (bacteria) and *Candida albicans* (yeast).

*Stevia rebaudiana* has been explored as an eco-friendly agent for NP synthesis, aligning with the concepts of green chemistry [13]. The nutrients in *S. rebaudiana* (e.g., carbohydrates, minerals, enzymes, vitamins, and antioxidants) act as reducing agents, stabilizers, and surfactants. Additionally, compounds like amine groups, water-soluble heterocyclic compounds, polyols, and proteins play key roles in reducing metal ions and stabilizing metal nanoparticles [17]. Although ZnO-NPs have been thoroughly studied for their antimicrobial activity against various bacterial strains [10,18,19], their application in targeting zoonotic MDR *E. coli* strains, particularly those found in retail fish markets, remains underexplored. Thus, the use of *S. rebaudiana* as a green, eco-friendly capping agent introduces a promising approach to ZnO-NP biosynthesis, offering a sustainable alternative that reduces environmental impact while potentially enhancing antimicrobial efficacy.

Therefore, this study aims to break new ground by developing and characterizing ZnO-NPs using *S. rebaudiana* as a promising and sustainable capping agent. By leveraging the unique characteristics of these green-synthesized ZnO-NPs, the research aims to unlock their potent antibacterial and antibiofilm capabilities. This study will also delve into the mechanistic pathways underpinning their antibacterial actions. This study may contribute by filling critical gaps in our understanding of ZnO-NPs’ bioactivity, and aspires to pioneer novel applications in food safety, providing groundbreaking strategies to combat zoonotic MDR bacteria.

## 2. Results and Discussion

### 2.1. Incidence and Characterization of E. coli in Marketed Fish

A total of 322 bacterial isolates were screened from 120 fish samples; among these, 153 strains were confirmed as *E. coli* using the VITEK^®^ 2 system (Marcy l’Etoile, France). The distribution of these confirmed *E. coli* strains was 39 (25.5%), 79 (51.6%), and 35 (22.9%) from *O. niloticus*, *S. pilchardus*, and *S. scombrus,* respectively. Therefore, the occurrence of *E. coli* was observed in 89 (74.2%) of the 120 fish samples. These findings revealed a lower incidence of *E. coli* contamination compared to previous studies. Sifuna et al. [20] found a 100% *E. coli* contamination rate in all tested fish samples (*n* = 60) from the Kenyan market, while Thampuran et al. [21] found *E. coli* in 66% of fish samples (*n* = 414) in Cochin, India. The presence of *E. coli* in retail fish products is a common issue, often resulting from poor sanitary practices, and poses a significant risk of enteric diseases to consumers, underscoring the potential health risks to consumers [3].

The resistance of 153 *E. coli* strains to 12 different antibiotic agents was assessed, as summarized in Table S1. The obtained data indicated that the *E. coli* strains exhibited high resistance to sulfamethoxazole-trimethoprim (SXT), chloramphenicol (CHL), and enrofloxacin (ENR), with resistance rates exceeding 81%, 75%, and 70%, respectively. Analysis of antibiotic resistance profiles (Table S2) revealed that 77.8% (119/153) of the *E. coli* strains were MDR. Table S2 further categorized these MDR strains into 22 distinct resistance patterns, with P7a, P8b, P9, P5, and P5b being the most prevalent (44.5%). To further investigate the characteristics of these resistance patterns, 1 MDR *E. coli* strain was randomly selected from each of the 22 identified DRPs for further analyses.

The investigation into the incidence of antibiotic-resistance genes in MDR *E. coli* strains revealed significant findings. Notably, the *tetA* gene was detected in 82% of the strains, suggesting a potential for tetracycline resistance. For β-lactam antibiotics, the prevalence of resistance genes was substantial, with 73% harboring the *bla*_TEM_ gene, 59% carrying *bla*_IMP-25_, 55% possessing *bla*_SHV_, and 41% each containing *bla*_IMP-7_ and *bla*_OXA-2_, resulting in 100% of the strains having at least one *bla* gene. Additionally, a high prevalence of aminoglycoside resistance determinants was observed; the *aadA* and *aac(3)-IV* genes were detected in 86% and 55% of *E. coli* strains, respectively. The study also identified virulence factors, revealing that 59% of the strains contained the *stx*2 gene associated with Shiga toxin production, while only 14% had the *stx*1 gene. Furthermore, the *hly*A and *hly*D genes were detected in 18% and 27% of the strains, respectively. Other virulence genes, *pap*A (59%), *agg*R (50%), *estI*b (50%), *afa* (36%), *elt*B (27%), and *eae*A (14%), were also present.

Similarly, Ribeiro et al. [22] reported the presence of the *eaeA* gene in 6.25% of *E. coli* strains, with *stx1* and *stx2* detected in 2.08% and 5.21% of tested strains, respectively. Similarly, Siddhnath et al. [23] found that 24% of Shiga toxin-producing *E. coli* (STEC) strains from fish carried at least one virulence gene (*stx1*, *stx2*, *eaeA*, and *ehlyA*), while Joseph et al. [24] linked STEC infections to severe illnesses, including hemorrhagic colitis and acute renal failure. Additionally, virulence factors (e.g., *hlyA* and *papC*) are often associated with resistance, particularly to third-generation cephalosporins, complicating therapeutic options [25]. α-hemolysin, a pore-forming toxin, significantly enhances *E. coli* pathogenicity by causing cell lysis and triggering inflammatory responses [26]. Other factors (e.g., *eaeA*) promote epithelial adhesion, while *aggR* supports biofilm formation, contributing to persistent infections [27]. These findings underscore the interplay between antibiotic resistance and virulence, reinforcing the need for comprehensive prevention and treatment strategies for MDR *E. coli* infections.

Identification and the constructed phylogenetic tree of the selected *E. coli* strains of cluster X (Figure S1) were performed using *16S rRNA* gene sequencing. Figure S2 showed that MFEC-NT-22 and MFEC-M-10 showed 100% identity with *Escherichia coli* TUM1586 (CP135712) and *Escherichia coli* ST95-32 (CP043950), respectively. MFEC-M-1 showed 98.58% identity to *Escherichia coli* PNUSAE147328 (CP143968). However, MFEC-NT-6, MFEC-S-34, and MFEC-S-31 showed 97.75, 97.74, and 97.67%, identity to *Escherichia coli* E138 (AP022351), *Escherichia coli* SCU-485 (CP053245), and *Escherichia coli* O157:H7 PNUSAE146743 (CP133128), respectively. MFEC-S-2 showed 96.72% identity with *Escherichia coli* 8523 (CP133106).

### 2.2. Characterization of Green Synthesized ZnO-NPs

The chemical components of *S. rebaudiana* leaves were detected using GC–MS analysis, which showed that 16 phytochemicals were identified in the tested samples (Table S3). Similarly, Raghu and Velayudhannair [28] stated that *S. rebaudiana* has high concentrations of fatty acids, carbohydrates, and secondary metabolites, which demonstrate therapeutic properties and antimicrobial activity against various pathogens. In this study, *S. rebaudiana* was utilized as a renewable and sustainable resource, aligning with green chemistry principles by replacing harmful reducing agents with its bioactive compounds. The synthesis process was energy-efficient, occurring under mild reaction conditions at ambient temperature, thus reducing energy consumption compared to conventional methods [12,29]. The bioactive compounds in *S. rebaudiana*, including antioxidants and water-soluble compounds (Table S3), acted as sustainable catalysts, facilitating the reduction of metal ions into ZnO-NPs without producing toxic byproducts. Additionally, agricultural-waste-derived *S. rebaudiana* was employed to achieve waste reduction while maintaining the high yield, biocompatibility, and purity of the synthesized ZnO-NPs. Compared to conventional synthesis patterns, this green synthesis approach eliminates the need for high-temperature reactions, specific chemical precursors, and hazardous reagents [15]. Detailed characterization results support the ecological advantages of this method, demonstrating its efficiency, sustainability, and alignment with green chemistry principles.

**Figure 1:**
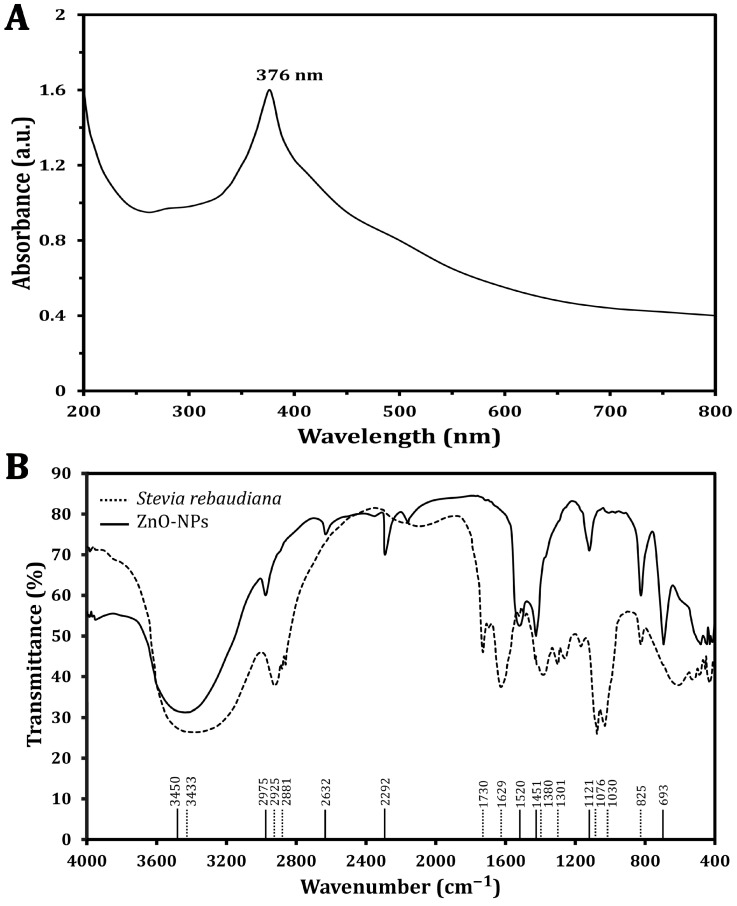
Characterization of the green synthesized ZnO-NPs using UV–vis spectra (**A**) and FTIR spectra analysis (**B**).

The synthesized ZnO-NP optical characteristics were analyzed, and a specific absorption peak was identified using a UV–visible spectrophotometer (Figure 1A), which revealed that the absorption peak characteristic of ZnO-NPs was found at a wavelength of 376 nm, confirming the formation of ZnO-NPs and highlighting the significant role of *S. rebaudiana* as a reducing agent to convert zinc ions into ZnO-NPs. The phytochemicals present in the plant extract, including polyphenols and flavonoids, are believed to play a pivotal role in this reduction process, leading to the formation of NPs with desirable properties. This method underscores the potential of utilizing natural resources for NP synthesis and aligns with sustainable practices in nanotechnology.

The FT-IR analyses of *S. rebaudiana* and the green synthesized ZnO-NPs using *S. rebaudiana* reveal distinct differences and some similarities in the peaks (Figure 1B). The broad peak at 3433 cm^−^^1^ in *S. rebaudiana* and 3450 cm^−^^1^ in the ZnO-NPs is indicative of O-H stretching vibrations [30]. This peak is associated with hydroxyl groups, suggesting the presence of alcohols or phenolics in the *S. rebaudiana* extract, which may be involved in the reduction and stabilization process of the ZnO-NPs [13]. However, peaks at 2925 cm^−^^1^ and 2881 cm^−^^1^ in *S. rebaudiana* correspond to C-H stretching vibrations of alkanes. The shift to 2975 cm^−^^1^ in ZnO-NPs, reflecting the interaction between the organic molecules in *S. rebaudiana* and the surface of the ZnO-NPs, is possibly due to the capping of NPs by organic molecules [13].

The peak at 1730 cm^−^^1^ in *S. rebaudiana* is attributed to C=O stretching vibrations, typical of carbonyl groups (e.g., aldehydes, ketones, or carboxylic acids) [31]. This peak is absent in the ZnO-NP spectrum, indicating that the carbonyl groups may have reacted during NP synthesis or were involved in the reduction process. Additionally, the peak at 1629 cm^−^^1^ in *S. rebaudiana* is attributed to C=C stretching vibrations, often found in aromatic rings or alkenes [32]. The shift to 1520 cm^−^^1^ in ZnO-NPs indicates an interaction between the aromatic compounds of *S. rebaudiana* and the ZnO-NPs, possibly due to the stabilization of NPs by π-electron clouds. The bending vibrations at 1380 cm^−^^1^ and 1301 cm^−^^1^ in *S. rebaudiana* are typical of C-H bonds in alkanes. The presence of a peak at 1451 cm^−^^1^ in ZnO-NPs suggests some modification in the bending vibrations due to the interaction of C-H-containing compounds with NPs’ surfaces [33].

The peaks at 1076 cm^−^^1^ and 1030 cm^−^^1^ in *S. rebaudiana* are associated with C-O stretching in alcohols, ethers, or esters [13,34]. The peak at 825 cm^−^^1^, present in both spectra, is attributed to C-H out-of-plane bending vibrations, typically in aromatic compounds. The persistence of this peak showed that some aromatic structures from *S. rebaudiana* remain intact and cap ZnO-NPs. However, the peak at 693 cm^−^^1^ in ZnO-NPs is characteristic of Zn-O stretching vibrations, confirming the formation of ZnO-NPs [35]. The FT-IR spectra indicated that the functional groups in *S. rebaudiana* are involved in the reduction, capping, and stabilization of ZnO-NPs [13]. Shifts in peak positions and the appearance/disappearance of certain peaks suggest strong interactions between the plant extract and ZnO, contributing to the effective synthesis of ZnO-NPs. The presence of peaks related to Zn-O bonding confirms the formation of the NPs, while the organic peaks indicate that the *S. rebaudiana* extract has provided a stabilizing matrix for the ZnO-NPs [13].

**Figure 2:**
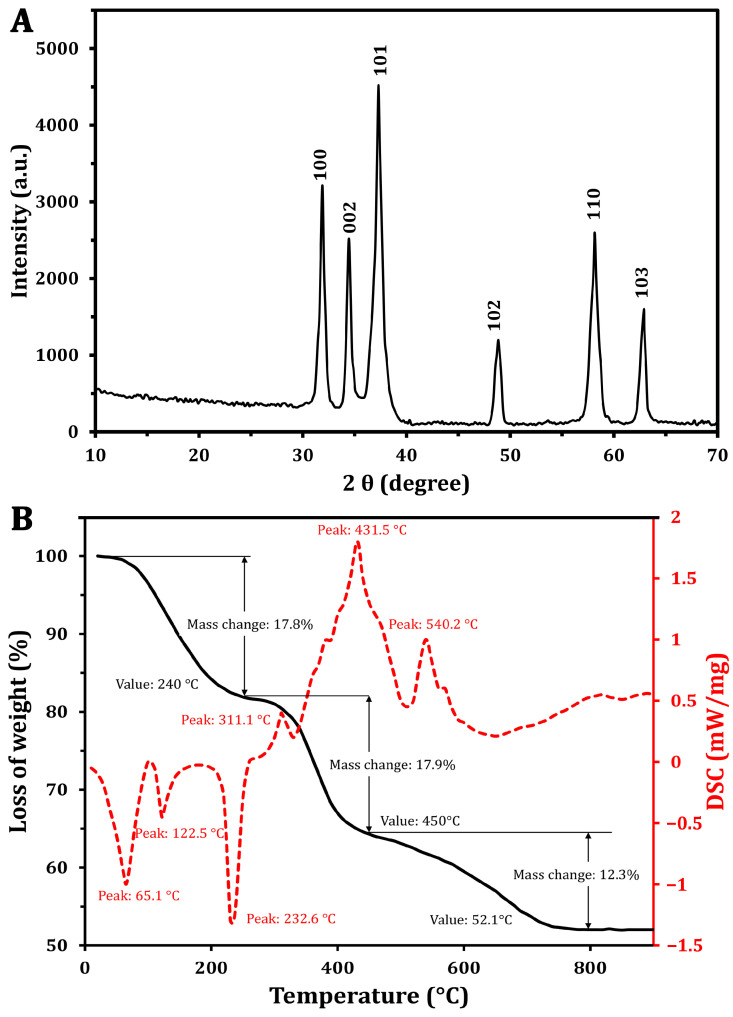
Characterization of the green synthesized ZnO-NPs using XRD (**A**) and TGA/DSC analysis (**B**).

To assess the crystalline structure of the synthesized ZnO-NPs, XRD analysis was performed (Figure 2A), which showed distinct and narrow peaks at various diffraction angles. These peaks are indicative of the high crystallinity and phase purity of the ZnO-NPs, aligning with findings reported by Khatami et al. [36]. The analysis identified specific Miller indices corresponding to the crystallographic planes (100), (002), (101), (102), (110), and (103), with peak positions measured at 31.87°, 34.44°, 37.29°, 48.8°, 58.13°, and 62.8°, respectively. The observed diffraction pattern closely matches the standard XRD data for hexagonal wurtzite ZnO (JCPDS No. 36-1451), confirming the successful synthesis of high-purity ZnO-NPs without detectable impurities. Furthermore, the average particle size of ZnO-NPs was calculated using the Debye–Scherrer equation (Table 1), resulting in an estimated size of approximately 12 nm. This calculation underscores the nanoscale dimensions of the synthesized particles, which are crucial for their potential applications in various fields.

The thermal degradation characteristics of the ZnO-NPs were analyzed through TGA and DSC. The TGA (Figure 2B) revealed distinct weight loss occurring in three distinct phases. Initially, a weight loss of 17.8% was found at 240 °C. As the temperature increased from 240 °C to 450 °C, an additional weight loss of approximately 17.9% was recorded [37]. Notably, in the range of 450 to 790 °C, a reversal in the weight loss trend was observed, resulting in a total weight loss of 48%, with a reduction of 12.3%. Importantly, no significant weight loss was detected at temperatures exceeding 752 °C, indicating the stabilization of NPs.

The DSC curve (Figure 2B) exhibited a complex thermal profile with several endothermic and exothermic peaks. One notable endothermic peak was observed at 125.5 °C, and began around 100 °C. This is attributed to the vaporization of water absorbed by the ZnO-NPs. The slight temperature shift from the typical 100 °C water vaporization point may be associated with the release of unstable wetting agent molecules that adhered to NP surfaces during synthesis [38]. Additionally, a peak at 232.6 °C likely indicates the transformation of a zinc precursor into zinc hydroxide, which may be an intermediate step in the ZnO-NP formation process [39]. The exothermic peak at 431.5 °C corresponds to the production of the final ZnO-NPs and the breakdown of any remaining organic materials. Similar findings were reported by Khatami et al. [36], who found a high thermal stability of the green synthesized ZnO-NPs using Stevia extract. However, the author found that a weight loss of 5.6% can be attributed to the thermal degradation of biomolecules (e.g., steviol glycosides).

These findings suggest that, although the XRD analysis confirmed ZnO-NPs’ crystallinity, additional high-temperature annealing may be necessary to achieve improved purification. The exothermic peak observed at 540.2 °C indicates the complete oxidation of zinc, resulting in the formation of the crystalline ZnO phase. The presence of a peak at 232.6 °C in the thermal analysis data suggests the transformation of a zinc-containing precursor into zinc hydroxide during the synthesis process [39]. This intermediate step may be part of the overall reaction pathway leading to the formation of the final ZnO-NPs.

**Figure 3:**
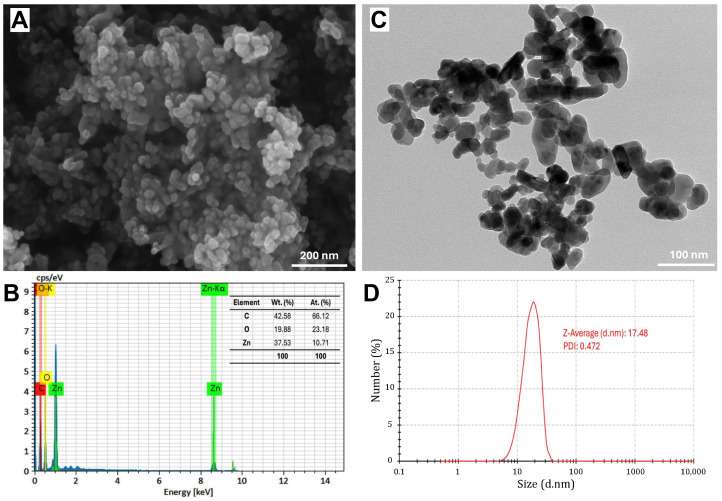
Characterization of green-synthesized ZnO nanoparticles using scanning electron microscopy (SEM) image (**A**), energy-dispersive X-ray spectroscopy (EDS) spectrum (**B**), transmission electron microscopy (TEM) image (**C**), and dynamic light scattering (DLS) analysis (**D**).

The SEM image reveals the structural morphology of the synthesized ZnO-NPs, showing a uniform distribution of rectangular, spherical, or semi-spherical particles (Figure 3A). Fiji software showed that the average size in SEM analysis was 24.8 nm. This was in agreement with Guirguis et al. [13], who found that the ZnO-NPs formed using *S. rebaudiana* extract showed semi-spherical particles with a particle size ranging from 10 to 90 nm. However, Khatami et al. [36] observed that ZnO-NPs displayed primarily rectangular morphology, accompanied by square, octagonal, and oval bar shapes. The SEM-EDS analysis confirmed the elemental composition of NPs, where Zn and O are the dominant components, with a weight percentage of 37.53% and 19.88%, respectively, corresponding to their atomic percentages of 10.71% and 23.18%. Additionally, the presence of carbon (42.58% by weight), as shown in Figure 3B, may be attributed to the organic residues from the green synthesis process and the stabilizing agents used during synthesis, as reported by Khatami et al. [36]. The TEM image also provides further insights into particle size and morphology (Figure 3C), revealing the presence of dispersed nanoparticles with an average size of 25.4 nm. The DLS analysis confirms the hydrodynamic diameter of the nanoparticles, with a Z-average size of 17.48 nm and a polydispersity index (PDI) of 0.472 (Figure 3D).

It is noteworthy to mention that XRD provides an estimation of the crystalline domain size, which can be comparatively smaller than the physical particle size observed via DLS, TEM, and SEM [40,41]. However, DLS measures the hydrodynamic diameter, which is typically larger due to the solvent layer surrounding the nanoparticles. However, DLS is sensitive to particle concentration, and the results can be varied due to multi-scattering effects or aggregation at high concentrations [42]. This aligns with the findings of Alam et al. [43], where biosynthesized AgNPs exhibited an average particle size of 11.5 nm by XRD, 85 nm by DLS, and 10–20 nm by TEM. The variations in the present study may arise from the fundamental differences in sample preparation and measurement techniques. Additionally, TEM and SEM may show relatively larger sizes (~25 nm) compared to DLS (~17.5 nm) due to minor aggregation due to solvent removal during sample preparation or the presence of residual organic stabilizers (42.58% carbon), attributed to particle aggregation.

The combination of SEM-EDS, TEM, and DLS analyses confirmed the successful synthesis of pure ZnO-NPs with nanoscale dimensions and a well-defined composition. Residual carbon from the green synthesis method may enhance nanoparticle stabilization. This characterization highlights the potential of ZnO-NPs in antimicrobial and biotechnological applications by providing insights into their morphology, size, and composition. *S. rebaudiana*, rich in functional biomolecules, facilitates zinc ion complexation and acts as a capping agent, stabilizing the nanoparticles and influencing their interactions in various applications [44].

### 2.3. ZnO-NPs’ Antibacterial and Antibiofilm Activity

The antibacterial activity of the synthesized ZnO-NPs was investigated against the selected MDR *E. coli* strains based on their possession of the most virulent and resistant genes. The IZ diameters increased with higher concentrations of ZnO-NPs, ranging from 8.2 ± 0.4 mm to 19.2 ± 1.6 mm, indicating the varying susceptibility of the MDR *E. coli* strains (Table 2). Statistical analysis using the Tukey test showed significant differences in the IZ diameters between different MDR strains and ZnO-NP concentrations (*p* < 0.05). The potent dose-dependent antibacterial activity of the green-synthesized ZnO-NPs against these MDR strains highlights their potential as effective antimicrobial agents for improving food safety and production.

MIC values ranged from 12.5 to 32 µg/mL, and MBC values ranged from 25 to 64 µg/mL, with lower values indicating a higher susceptibility of the MDR *E. coli* strains to ZnO-NPs. The highest MIC against MFEC-M-10 (MDR *E. coli* strains isolated from Atlantic mackerel) was 32 µg/mL, while the lowest was 12.5 µg/mL against MFEC-NT-6, MFEC-S-34, and MFEC-S-2 (MDR *E. coli* strains isolated from Nile tilapia and European sardine). The differences in MIC and MBC values among the *E. coli* strains reflect their varying levels of resistance, which could be related to their genetic composition and virulence factors. Similar results were obtained by Ahmad et al. [45], who found that ZnO-NPs acting as potential antibacterial agents greatly reduced the growth of different pathogenic bacterial strains, with IZ varying from 16 to 21 mm with MIC varying from 15.6 to 125 µg/mL and MBC from 62.5 to 250 µg/mL.

Additionally, ZnO-NPs showed effective biofilm inhibition against MDR *E. coli* strains. Table 3 showed a dose-dependent inhibition of biofilm formation across all tested strains. Specifically, biofilm inhibition percentages significantly increased with increasing ZnO-NP concentration, from 50 µg to 250 µg. Among the tested strains, MFEC-NT-22 showed the highest inhibition at 250 µg (86.4%), indicating higher susceptibility to ZnO-NPs, while MFEC-S-2 was comparatively more resistant, with a maximum inhibition of 75.3% at the same concentration.

Biofilms are organized communities of bacteria enveloped in a self-generated extracellular polymeric substance (EPS), significantly enhancing their resistance to traditional antibiotics and immune system defenses [46]. The EPS matrix acts as a protective barrier around the biofilm cells, effectively shielding them from various bacteriostatic and bactericidal agents. This matrix impedes the penetration of these substances and prevents them from adhering to the bacterial cell walls, thereby contributing to the biofilm’s resilience against antimicrobial treatments [47]. The unique properties of ZnO-NPs, including their antibacterial activity and ability to disrupt biofilm architecture due to their small size and large surface area, make them an attractive option for medical and industrial applications [48].

ZnO-NPs exhibit intrinsic antibacterial properties because of their capability to generate reactive oxygen species (ROS) upon exposure to light or moisture. These ROS can damage bacterial cell membranes and DNA, leading to cell death. Rosenberg et al. [49] found that ZnO-NP MIC can effectively reduce the viability of *E. coli* cells, thereby limiting their ability to form biofilms. ZnO-NPs prevent the initial adhesion of *E. coli* and disrupt established biofilms. Husain et al. [18] indicated that ZnO-NPs can reduce biofilm formation by significant percentages, with higher concentrations leading to greater inhibition. For instance, sub-MIC concentrations of ZnO-NPs have been reported to decrease biofilm formation by over 60% [18].

The effectiveness of ZnO-NPs can vary based on environmental conditions and the bacterial strain involved. ZnO-NPs exhibit selective antibiofilm properties, showing greater efficacy in oligotrophic conditions (nutrient-poor environments) compared to nutrient-rich media [12,50]. Therefore, biosynthesized ZnO-NPs could represent a promising candidate for combatting biofilm-associated infections caused by *E. coli*. However, further research and development are necessary to fully understand their mechanisms and optimize their application in real-world scenarios.

### 2.4. ZnO-NPs’ Antibacterial Mechanism

**Figure 4:**
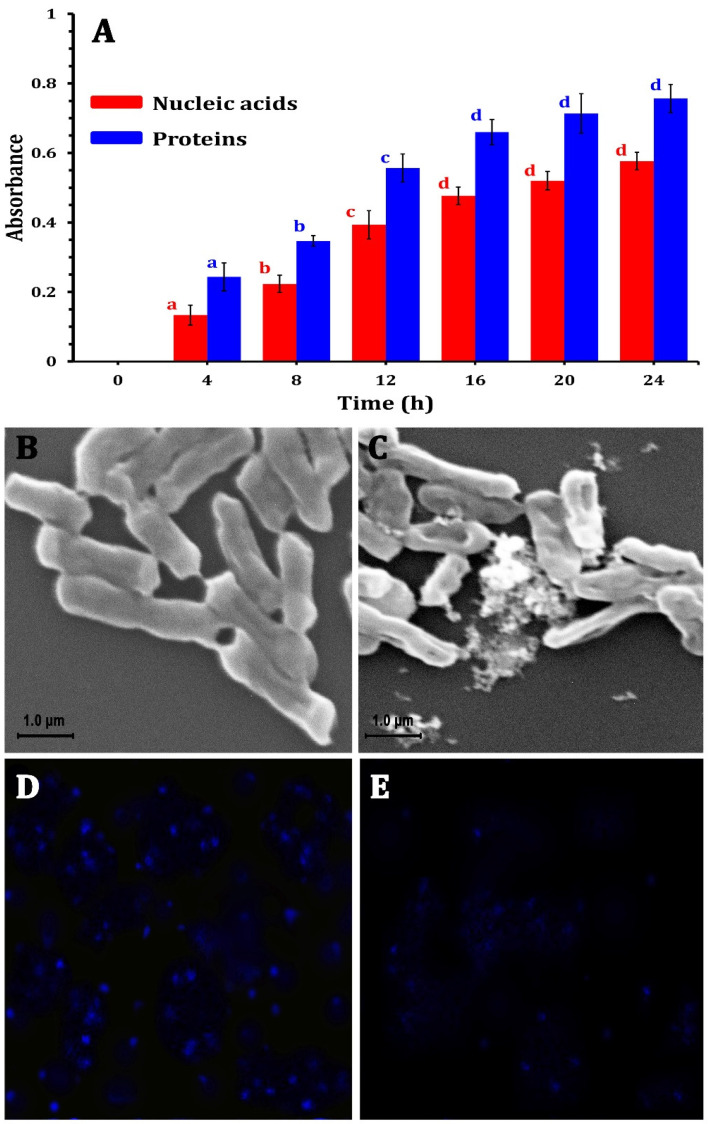
The mechanism of ZnO-NPs against MDR *E. coli* strains by releasing nucleic acids and proteins into the medium after treatment (**A**), SEM photomicrographs of *E. coli* bacterial cells without treatment (**B**) and after treatment using ZO-NPs (**C**), changes in bacterial DNA content by laser scanning confocal microscopy for *E. coli* cells showing DNA without ZnO-NPs treatment (**D**), and treated *E. coli* cells (**E**).

The ability of the ZnO-NPs to disrupt the bacterial cell membrane integrity was evaluated by measuring the release of intracellular components (e.g., proteins and nucleic acids) into the culture medium. Figure 4A shows the absorbance of the supernatant collected from the ZnO-NP-treated samples, revealing a potential increase compared with the control with a longer exposure time. This substantial rise in absorbance at 260 nm and 280 nm indicated a marked release of nucleic acids and proteins into the medium, suggesting that the ZnO-NPs could compromise the structural integrity of the bacterial cell membranes. This compromised membrane integrity led to the uncontrolled efflux of vital macromolecules from the bacterial cells, further demonstrating the potent antimicrobial activity of ZnO-NPs against the tested bacterial strain, causing a disruptive impact on bacterial cell integrity. The integrity of the cell membrane plays a major role in determining its capacity to develop normally. The statistical analysis revealed that protein and nucleic acid release significantly (*p* < 0.05) increased with 12 h of incubation, and after that increased non-significantly until 24 h (Figure 4A). In this context, Yang et al. [51] and Agarwal et al. [52] reported that bacterial cell membrane permeability increases when NPs enter bacterial cells due to the damage to the lipid layer due to the oxidative effect of ROS.

On the other hand, to investigate the morphological changes induced by ZnO-NPs, SEM analyses were conducted, revealing that the morphology of the untreated bacterial cells was consistently regular with a smooth surface (Figure 4B). However, after 24 h of ZnO-NP treatment at their respective MICs, the bacterial cells displayed an irregularly folded bacterial surface (Figure 4C). This was accompanied by significant cellular damage, evidenced by the aggregation and fragmentation of the treated cells. These observations underscore the severe structural alterations that occur in bacterial cells due to ZnO-NP exposure.

Moreover, the LSCM analysis revealed substantial differences in bacterial DNA content in *E. coli* cells treated with ZnO-NPs in comparison with control groups. ZnO-NPs had a significant influence on the quantity of DNA. The control sample (Figure 4D) showed higher fluorescence than the treated bacteria. The interaction between the nano-bio hybrid system and bacterial DNA might cause DNA damage (Figure 4E). These impacts can disrupt essential biological processes such as DNA replication, transcription, and repair, resulting in bacterial growth suppression or cell death. Agyemang et al. [53] revealed similar findings, stating that antibacterial agents alter the permeability of the cell cytoplasm, disrupting both DNA and ATP production processes and leading to microbial cell death.

**Figure 5:**
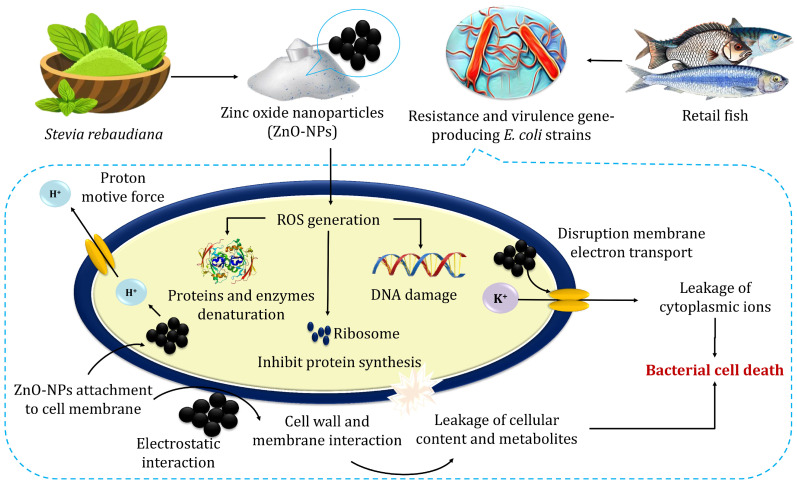
The possible mechanisms for the antibacterial activity of zinc oxide nanoparticles.

The proposed antibacterial mechanism of ZnO-NPs indicated their impact on bacterial cell walls and DNA (Figure 5). Atapakala et al. [12] emphasized that lipoteichoic and teichoic acids in bacterial cell walls play a vital role in binding Zn^2+^ ions, facilitating their transport into bacterial cells and particularly enhancing antibacterial effects. The structural differences between Gram-positive and Gram-negative bacteria influence ZnO-NP interactions. For instance, *E. coli*’s thin peptidoglycan layer and porins enable the passive diffusion of ZnO-NPs, leading to intracellular accumulation [52]. Additionally, Agarwal et al. [52] stated that the antibacterial mechanisms of green-synthesized ZnO-NPs exhibit enhanced bacterial binding due to their high surface area and multiple ligands. Antibacterial effects stem from cell membrane disruption, Zn^2+^ release, and subsequent ROS generation, causing DNA damage, protein and lipid oxidation, and metabolic enzyme inhibition. ZnO-NPs also inhibit zinc efflux pumps, increasing intracellular zinc concentrations. The efficacy of ZnO-NPs depends on their size, shape, and concentration, positioning them as promising antibacterial agents across diverse industries, including agriculture, food, cosmetics, and pharmaceuticals, offering potential solutions against multidrug-resistant bacteria [52].

## 3. Materials and Methods

### 3.1. Sample Collection and E. coli Isolation

A total of 120 fish, comprising 40 each of *O. niloticus*, *S. pilchardus,* and *S. Scombrus* samples, were randomly purchased from a local fish market in Zagazig City, Egypt. The samples were immediately transported in aseptically polyethylene bags to the laboratory and kept in the refrigerator at (0–4 °C). The sample collection was conducted under the guidelines and regulations established by the Faculty of Veterinary Medicine, Cairo University Council.

**Figure 6:**
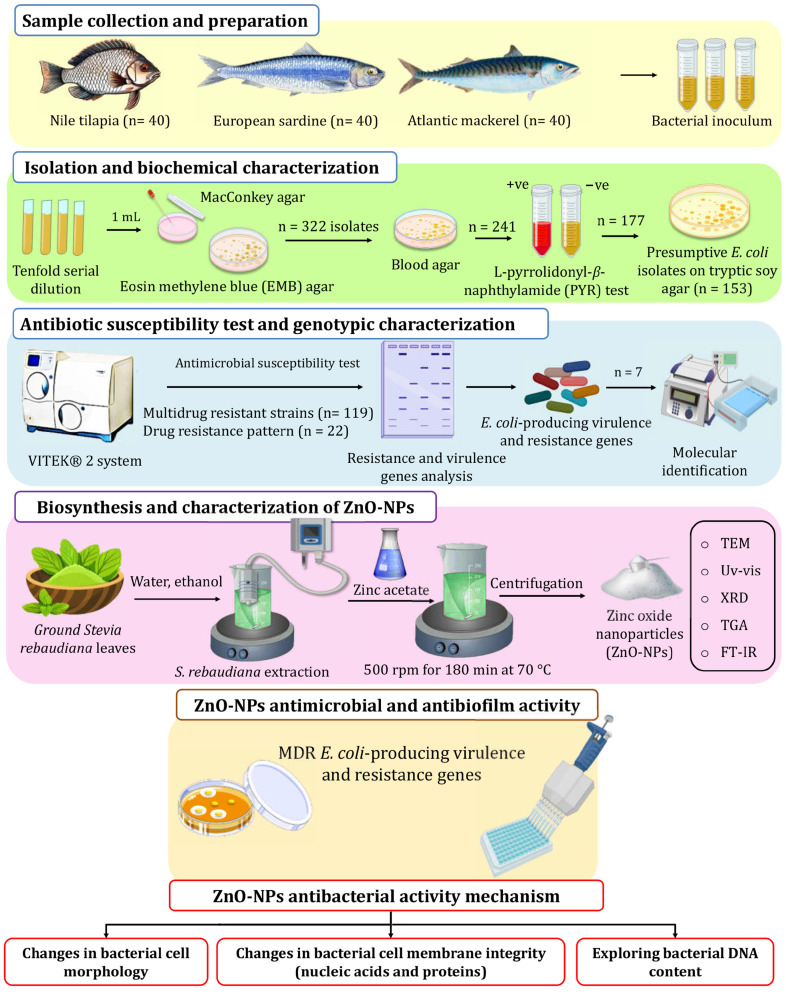
The experimental setup used in this study for screening isolation, characterization of *E. coli* from retail fish samples, and ZnO-NP biosynthesis using *S. rebaudiana*.

For *E. coli* screening and isolation, fish skin, gills, and gut were swabbed and inoculated into l0 mL peptone, and then incubated overnight to prepare a tenfold serial dilution (10^−3^–10^−7^). A volume of 1.0 mL from the prepared dilution was plated on MacConkey agar and Eosin Methylene Blue (EMB) agar, and then incubated for 48 h at 37 °C. *E. coli*-suspected colonies were picked according to their phenotypic properties, as shown in Figure S3 [54]. Suspected colonies were then transferred to blood agar plates (BAP) to test for β-hemolysis. Positive isolates were further tested using a PYR test (Figure 6). A colony from BAP was chosen for microscopic investigation. The biochemical identification of Gram-negative bacteria was carried out with the VITEK^®^ 2 system (Marcy l’Etoile, France) with a GNId card (Marcy l’Etoile, France) to confirm the presumptive *E. coli* isolates.

### 3.2. Antibiotic Susceptibility Testing

The bioMérieux VITEK^®^ 2 Compact system (Marcy l’Etoile, France) was utilized to identify multidrug-resistant (MDR) strains. This process involved Gram-Negative Identification (GNI) and antibiotic susceptibility testing (AST) card utilization, following the manufacturer’s instructions. The VITEK^®^ 2 (Marcy l’Etoile, France) Compact system enabled the identification and evaluation of the antibiotic susceptibilities of the tested strains. A range of antibiotic agents from various categories was employed in the study, as shown in Table S4, including Amikacin (AMK), Gentamicin (GEN), Cefalexin (CFX), Cefpodoxime (CEP), Chloramphenicol (CHL), Marbofloxacin (MAR), Enrofloxacin (ENR), Imipenem (IMI), Polymyxin B (POL), Tetracycline (TET), Piperacillin (PIP), and Trimethoprim-sulfamethoxazole (SXT). Results were interpreted following CLSI [55] guidelines. MDR was defined as resistance to at least three tested antibacterial agents [56]. The multiple antibiotic resistance (MAR) indices were evaluated following Ayandele et al. [57].

### 3.3. Molecular Characterization

The polymerase chain reaction (PCR) assay was applied to identify drug resistance genes and virulence factors. The resistance genes tested included aminoglycoside resistance genes (*aad*A, *aac*(3)-IV), extended-spectrum β-lactamase (*bla*_OXA-2_), β-lactamase genes (*bla*_TEM_, *bla*_IMP-7_, *bla*_IMP-25_, and *bla*_SHV_), and tetracycline resistance genes (*tet*A). Virulence factor genes included heat-stable enterotoxin (*elt*B), afimbrial adhesion (*afa*), aggregative virulence regulator (*agg*R), cytolytic protein toxin (*hly*D), enterohemolysin (*hly*A), heat-labile enterotoxin (*estI*b), intimin (*eae*A), P fimbria (*pap*A), and Shiga toxins 1 and 2 (*stx*1 and *stx*2).

PCR was conducted in a 25 μL reaction mixture containing 1 μL of template DNA, which was added to a final volume of 25 μL PCR mixture comprising 12.5 μL of Taq PCR Master Mix (Fermentas, Altrincham, UK; Catalog No. K0171), including, 1× PCR buffer (50 mM KCl and 10 mM Tris–HCl, pH 8.3), 1.5 mmol/L MgCl_2_, 0.15 mmol/L dNTP, and 1.25 IU Taq DNA polymerase, 1 μL of 0.8 μmol/L each primer, and 9.5 μL of sterile distilled water [58]. The amplification process began with an initial denaturation at 95 °C for 10 min, followed by 35 cycles of denaturation at 95 °C for 45 s, annealing at primer-specific temperatures for 30 s, and extension at 72 °C for 45 s. A final elongation step at 72 °C for 10 min ensured the complete extension of the PCR products. The amplicons were then visualized using gel electrophoresis on a 1.2% agarose gel stained with ethidium bromide, running at 100 V for 30 min.

For the molecular identification of *E. coli* strains, the *16S rRNA* gene was targeted. Genomic DNA was extracted from each strain using the TaKaRa MiniBEST Kit Ver.3.0 (TakaRa, Kyoto, Japan). The *16S rRNA* gene was amplified using 27F and 1492R (universal primers), as described by Sano et al. [59]. PCR was carried out on a Bio-Rad S1000™ Thermal Cycler (Bio-Rad, Hercules, CA, USA) under the conditions specified in Table S5 [54,60,61,62,63,64,65,66,67]. After amplification, products were separated by electrophoresis on a 1.2% (*w*/*v*) agarose gel containing 0.1 μg/mL ethidium bromide in 1× TAE buffer for 30 min at 100 V, and the bands were visualized under UV light. The purified products were sequenced at Sangon Biotech (Shanghai, China). Sequence similarity was analyzed and aligned using the GenBank database tools (http://www.ncbi.nlm.nih.gov/BLAST/ (accessed on 7 August 2024)).

### 3.4. ZnO-NP Synthesis

As shown in Figure 6, ZnO-NPs were synthesized in an eco-friendly way using the auto-combustion method, where zinc nitrate was used as the precursor material *S. rebaudiana* ground leaves were obtained from and identified by the School of Agricultural Engineering, Jiangsu University, and employed as a reducing and stabilizer agent. The extraction of *S. rebaudiana* extract followed the method described by Essa et al. [68], employing ultrasound-assisted extraction and subsequent ethanol (96% *v*/*v*) evaporation. For the green synthesis of ZnO-NPs, zinc acetate solution (0.1 M) in deionized water (100 mL) was heated to 35 °C at 500 rpm. Then, the *S. rebaudiana* extract solution was added dropwise to the zinc acetate solution in a ratio of 5/3 *v*/*v* and the pH value was adjusted to 10 using NaOH solution (4 M). Heating continued to 70 °C, with a constant temperature maintained under stirring at 500 rpm for 180 min. Subsequently, the mixture underwent centrifugation to remove the supernatant, followed by two washes to eliminate impurities. The resulting precipitate was dried at room temperature and ground into a fine powder for characterization and subsequent applications.

#### 3.4.1. Phytochemical Analysis of *S. rebaudiana*

Powdered *S. rebaudiana* leaves (20 g) were extracted with 250 mL of ethanol at 65 °C for 24 h using a Soxhlet extractor. The solvent was evaporated using a rotary vacuum evaporator and subjected to GC-MS analysis using gas chromatography with flame ionization detection (GC-FID, Shimadzu GC-2010 Plus, Kyoto, Japan) on a FAMEWAX (USP G16) fused silica capillary column (30 m × 0.32 mm × 0.25 mm). Helium was used as the carrier gas, with a flow rate of 30 mL/min. The column temperature was set at 40 °C and increased to 150 °C at 5 °C/min, and then to 200 °C at 10 °C/min and to 240 °C at 2 °C/min. The bioactive compounds were identified based on retention times using the National Institute of Standards and Technology (NIST) database for mass spectrum interpretation.

#### 3.4.2. Characterization of the Green Synthesized ZnO-NPs

The optical properties of the synthesized ZnO-NPs were investigated using a Shimadzu UV-1800 visible spectrophotometer in the wavelength range of 200–800 nm. However, Fourier-transform infrared (FT-IR) spectroscopy was employed to detect the functional groups in ZnO-NPs using a Perkin Elmer FT-IR spectrophotometer-RXI. The FTIR analysis was conducted in the wavenumber range from 400 to 4000 cm^−1^. ZnO-NPs’ crystalline structure and purity were detected using a Miniflex 600 X-ray diffractometer (Rigaku, Tokyo, Japan) with Cu-Kα radiation (*λ* = 0.154056 Å) at 40 kV and 30 mA, with a scan rate of 2θ = 5°/min. The XRD pattern provides information about the crystallographic planes and allows for the calculation of the average crystallite size using the following Debye–Scherrer equation:

(1) $$Crystallite\;size=\frac{K\lambda }{\beta cos\theta }$$

where *K* = 0.95, *β* is the full width at half maximum (FWHM) of the diffraction peak, and *θ* is the Bragg diffraction angle.

The thermal characteristics and weight loss of the synthesized NPs were explored using thermogravimetric analysis (TGA) with a Shimadzu TGA-50 instrument. Differential scanning calorimetry (DSC) analysis was carried out using a Shimadzu DSC-60A under a nitrogen environment, with a heating rate of 10 °C/min. The morphological characteristics and particle size of the synthesized NPs were characterized using a Scanning Electron Microscope-Energy Dispersive X-ray (SEM-EDS) Regulus Hitachi, Hitachi H-800 (Tokyo, Japan), and transmission electron microscopy (TEM) with an accelerating voltage of 100 kV. To facilitate visual interpretation, Fiji software (V. 1.54g) was used to detect the average size of nanoparticles [69]. Moreover, ZnO-NPs’ diameter and the polydispersity index (PDI) were detected by dynamic light scattering (DLS) analysis using Zetasizer Nano ZS90 (Malvern, UK), following Yang et al. [70].

### 3.5. ZnO-NPs’ Bioactivity Against Pathogenic MDR E. coli Strains

#### 3.5.1. Antibacterial Activity

The antibacterial activity of the green synthesized ZnO-NPs was evaluated against selected MDR *E. coli* strains using the disk diffusion method [12]. Briefly, bacterial cultures were inoculated into the nutrient broth medium (5 mL) and incubated in a shaking incubator until the culture density reached 0.5 McFarland units (~1.5 × 10^8^ colony-forming units). The bacterial inoculum was then evenly spread onto nutrient agar plates. Sterile Whatman No. 1 filter paper disks were placed onto the agar surface, after inoculation with the prepared ZnO-NP suspensions (5 μg to 20 μg), and incubated for 48 h at 37 °C. Following incubation, to detect ZnO-NP antibacterial potential, the inhibition zone (IZ) surrounding each disk was measured in millimeters. The Minimum Inhibitory Concentration (MIC) was detected following Guirguis et al. [13]. Several concentrations of ZnO-NPs were prepared for the study. Subsequently, 2 μL of bacterial inoculum was added to each well of a 96-well microplate. The plates were then incubated at 37 °C for 24 h. MIC was defined as the lowest concentration of ZnO-NPs that effectively inhibited visible bacterial growth following the incubation period. However, the Minimum Bactericidal Concentration (MBC) was determined as the lowest concentration of ZnO-NPs that completely prevented bacterial growth.

#### 3.5.2. Antibiofilm Activity

The green synthesized ZnO-NPs’ antibiofilm activity was evaluated using a modified method described by Mohanta et al. [71]. Briefly, nutrient broth (10 mL) was inoculated with a loopful of test *E. coli* strains from an overnight nutrient agar culture. The prepared culture was diluted to an OD_600_ nm of 1.0 to be inoculated in the 96-well microtiter plate. Each well contained 150 μL of the bacterial culture and 10 μL of ZnO-NP. The contents were thoroughly mixed and incubated for 24 h at 37 °C. A sterile broth mixed with ZnO-NPs served as the blank control.

Following the incubation period, the non-adherent cells and any floating material were removed from the wells. Thereafter, each well was washed three times with 200 μL of saline solution to eliminate any remaining non-adherent cells, and the wells were allowed to air-dry for 30 min. To fix the adherent biofilms, a solution of 2% sodium acetate was applied, and the biofilms were subsequently stained with 0.1% crystal violet for 10 min. After staining, excess dye was washed away with saline, and the plates were air-dried to prepare for quantification. To quantify biofilm formation, the stained biofilms were solubilized by adding 200 μL of 95% ethanol and incubating for 10 min. The solubilized biofilm absorbance was measured at an optical density of 620 nm using a microplate reader (Biorad 680, Berkeley, CA, USA). The inhibition of biofilm formation percentage was assessed according to the following equation:

(2) $$Biofilm\;inhibition(\%)=\frac{\left(Ac-At\right)\times 100}{Ac}$$

where At is the absorbance of the treated sample and Ac is the absorbance of the control.

### 3.6. ZnO-NP Mechanism as an Antibacterial Agent

#### 3.6.1. Changes in Bacterial Morphological Characteristics

The morphological changes in bacteria upon exposure to ZnO-NPs were assessed using scanning electron microscopy (SEM; JEOL-IT500A, Tokyo, Japan). A bacterial culture with an optical density of 1.0 OD_600_ nm was prepared, and ZnO-NPs were added to their MIC. After the mixture was incubated at 37 °C for 12 h, the samples were centrifuged for 10 min at 8000 rpm to collect the cell pellets. Thereafter, the pellets were washed with 0.1 M phosphate buffer (pH 7.4) to remove residual media. To fix the cells, they were treated with 2.5% glutaraldehyde at 4 °C for 60 min, followed by an additional fixation period of 180 min at room temperature, as described by Zhang et al. [72]. After fixation, the cell pellets were centrifuged for 10 min at 8000 rpm. The collected pellets underwent dehydration through a series of ethanol solutions, with increasing concentrations from 30% to 100%. Following dehydration, the cells were coated with gold for 2 min to enhance conductivity.

#### 3.6.2. Cell Membrane Integrity

The cell membrane integrity was detected by assessing the release of proteins and nucleic acids (i.e., cellular components) after bacterial cell treatment with ZnO-NPs. The bacterial culture was incubated at 37 °C, and a sample with 1.0 OD_600_ nm was treated with different concentrations of ZnO-NPs [72]. At different time intervals during a 24 h period, the cell suspension was centrifuged for 5 min at 8000 rpm, and the supernatants were measured at 260 and 280 nm for nucleic acids and proteins, respectively. As the control, the untreated bacterial cells were used against a phosphate-buffered saline (PBS) blank.

#### 3.6.3. Changes in Bacterial Cell DNA Content

TCS SP5 II Laser scanning confocal microscopy (LSCM; Leica, Wetzlar, Germany) was used to assess variations in bacterial DNA content. After 24 h of incubation at 180 rpm and 37 °C, the bacterial suspension treated with ZnO-NPs was combined with an equivalent amount of 4′,6-diamidino-2-phenylindole (DAPI, Roche Diagnostics GmbH’s, Mannheim, Germany) solution with a concentration of 10 µg/mL. A drop of this mixture was placed on a microscope slide and left in the dark for 10 min. The control solution was prepared without ZnO-NPs. The peak excitation and emission wavelengths were 364 and 454 nm, respectively [73].

### 3.7. Statistical Analysis

The statistical analysis of the obtained data was visualized by GraphPad Prism version (8.0.2). To compare the differences between groups, an independent *t*-test was employed. PC-ORD version 7 was utilized to perform a two-way hierarchical cluster analysis. The analysis was conducted using the Sorensen (Bray–Curtis) method for group linkage, which enabled the clustering of similar groups based on their characteristics. ANOVA (Analysis of Variance) and Tukey’s post hoc test were applied for multiple comparisons.

## 4. Conclusions

The escalating challenge of antibiotic resistance necessitates the development of alternative strategies to improve fish health and ensure consumer safety. This study successfully synthesized and characterized ZnO-NPs using *Stevia rebaudiana* as a sustainable reducing agent, adhering to green chemistry principles. The eco-friendly approach underscores the potential of ZnO-NPs in combating MDR *E. coli* strains, which are a major public health threat associated with retail fish markets, while aligning with global sustainability goals. The green-synthesized ZnO-NPs exhibited high purity and distinctive morphologies, with shapes ranging from semi-spherical to hexagonal. These nanoparticles demonstrated dose-dependent antibacterial activity against MDR *E. coli* strains, significantly reducing biofilm formation and pathogenicity. The findings highlight the efficiency of eco-friendly ZnO-NPs as potent antibacterial agents with promising applications in food safety, aquaculture, and other sectors. This study provides a foundation for future research to elucidate the antibacterial mechanisms of green-synthesized ZnO-NPs and explore their broader applicability across diverse domains, potentially advancing public health and sustainable practices.

## Figures and Tables

**Table 1:** ZnO-NP size assessment by Debye–Scherer equation.

Position (°2 θ)	Planes	FWHM Left (°2 θ)	Lattice Strain (ε)	Size (nm)
31.87	100	0.62174	0.0016	12.41
34.44	002	0.52491	0.0012	14.68
37.29	101	0.94222	0.002	8.19
48.8	102	0.57847	0.001	13.32
58.13	110	0.89517	0.0012	8.68
62.8	103	0.58732	0.0007	13.18
Average FWHM		0.691638	Average size	11.74

**Table 2:** ZnO-NP antibacterial activity in the zone of inhibition against various bacteria.

Strain Code	ZnO-NPs and IZ (mm) *				MIC (µg/mL)	MBC (µg/mL)
	5 µg/Disk	10 µg/Disk	15 µg/Disk	20 µg/Disk		
MFEC-S-31	10.5 ± 0.5 ^abc **^	13.7 ± 0.7 ^a^	14.6 ± 1.0 ^ab^	16.5 ± 1.5 ^ac^	25	50
MFEC-NT-6	10.3 ± 0.8 ^abc^	13.1 ± 1.1 ^a^	15.3 ± 1.8 ^a^	17.7 ± 1.2 ^a^	12.5	25
MFEC-S-34	11.5 ± 0.5 ^b^	13.5 ± 1.0 ^a^	15.5 ± 1.5 ^a^	18.3 ± 1.0 ^a^	12.5	25
MFEC-M-1	8.2 ± 0.4 ^c^	10.5 ± 1.1 ^b^	14.6 ± 0.8 ^ab^	16.6 ± 0.8 ^ac^	25	50
MFEC-S-2	9.6 ± 0.6 ^abc^	11.2 ± 1.2 ^b^	16.1 ± 1.1 ^a^	19.2 ± 1.6 ^b^	12.5	25
MFEC-M-10	9.3 ± 0.6 ^abc^	10.8 ± 0.7 ^b^	12.9 ± 0.9 ^b^	14.5 ± 1.0 ^c^	32	64
MFEC-NT-22	11.1 ± 1.0 ^ab^	12.5 ± 0.5 ^a^	14.2 ± 0.7 ^ab^	16.5 ± 0.5 ^ac^	25	50
*p*-value	0.905	0.809	0.876	0.837		

* IZ, Zone of inhibition; MBC, minimum bactericidal concentration; MIC, minimum inhibitory concentration. ** Means with the same letters in the same column show an insignificant difference.

**Table 3:** Antibiofilm activity of ZnO-NPs against resistant and virulent gene-producing *E. coli*.

Strain Code	Biofilm Inhibition (%)					
	0 µg	50 µg	100 µg	150 µg	200 µg	250 µg
MFEC-S-31	0.0 ± 0.0 ^a *^	19.1 ± 1.1 ^ac^	41.5 ± 2.6 ^a^	64.1 ± 1.7 ^a^	70.0 ± 2.7 ^a^	82.4 ± 2.5 ^a^
MFEC-NT-6	0.0 ± 0.0 ^a^	21.1 ± 0.8 ^abc^	43.2 ± 2.2 ^ad^	63.5 ± 2.0 ^a^	80.3 ± 1.6 ^b^	82.6 ± 3.1 ^a^
MFEC-S-34	0.0 ± 0.0 ^a^	18.5 ± 0.7 ^ac^	40.1 ± 1.8 ^a^	63.4 ± 2.1 ^a^	79.2 ± 2.2 ^b^	81.3 ± 2.2 ^a^
MFEC-M-1	0.0 ± 0.0 ^a^	22.3 ± 1.5 ^ac^	43.1 ± 2.5 ^ad^	65.5 ± 1.5 ^a^	81.3 ± 2.3 ^b^	84.3 ± 1.7 ^ac^
MFEC-S-2	0.0 ± 0.0 ^a^	17.4 ± 1.3 ^c^	28.6 ± 1.4 ^b^	48.6 ± 1.6 ^b^	65.3 ± 1.5 ^c^	75.3 ± 2.4 ^b^
MFEC-M-10	0.0 ± 0.0 ^a^	19.6 ± 0.6 ^ac^	33.4 ± 1.5 ^c^	52.6 ± 1.4 ^b^	70.4 ± 3.1 ^a^	80.3 ± 2.0 ^a^
MFEC-NT-22	0.0 ± 0.0 ^a^	25.2 ± 1.4 ^b^	46.1 ± 2.4 ^d^	66.4 ± 2.1 ^a^	82.6 ± 2.8 ^b^	86.4 ± 3.4 ^c^
*p*-value	0.982	0.961	0.996	0.804	0.902	0.982

* Means with the same letters in the same column show an insignificant difference.

## Data Availability

The data presented in this study are available upon request from the corresponding author.

## Supplementary Materials

The following supporting information can be downloaded at https://www.mdpi.com/article/10.3390/molecules30040768/s1, Figure S1. Clustering analysis two-way dendrogram of pathogenic MDR *E. coli* strains using Sorensen methods for distance; Figure S2. Neighbor-joining tree constructed from *16S rRNA* gene sequences, illustrating the phylogenetic placement of selected pathogenic MDR *E. coli* strains among closely related taxa; Figure S3. *E. coli* identification scheme; Table S1. The susceptibility of *E. coli* strains (*n* = 153) to antimicrobial agents; Table S2. The drug resistance patterns of MDR *E. coli* strains; Table S3. Phytochemical composition of *S. rebaudiana* using GC–MS; Table S4. Antimicrobial agents and zone interpretive chart based on CLSI guidelines; Table S5. Primers used for PCR amplification.
