# Supplementary material for: Antibacterial and Antibiofilm Activity of Green-Synthesized Zinc Oxide Nanoparticles Against Multidrug-Resistant Escherichia coli Isolated from Retail Fish
Source: Molecules. 2025 Feb 7;30(4):768. doi: 10.3390/molecules30040768 (PMC11858201; doi:10.3390/molecules30040768)
Supplement: Supplementary file 1 [file molecules-30-00768-s001.zip › molecules-3439722-supplementary.pdf]

Supplementary Data

Antibacterial and Antibiofilm Activity of Green-Synthesized Zinc Oxide Nanoparticles Against Multidrug-Resistant *Escherichia coli* Isolated from Retail Fish

Mohamed Tharwat Elabbasy[1], Rasha M. El Bayomi[2], Esraa A. Abdelkarim[2*], Abd El-Salam E. Hafez[2], Mohamed S. Othman[3], Mohamed E. Ghoniem[4], Mai A. Samak[1], Muteb H. Alshammari[5], Fahad Awwadh Almarshadi[6], Tamer Elsamahy[7*], Mohamed A. Hussein[2]

1. Department of Pathology, College of Medicine, University of Ha'il, Ha'il, Saudi Arabia.
2. Food Control Department, Faculty of Veterinary Medicine, Zagazig University, Zagazig, 44519, Egypt.
3. Department of Biochemistry, College of Medicine, University of Ha'il, Ha'il, Saudi Arabia.
4. Department of Internal Medicine, College of Medicine, University of Ha'il, Ha'il 2240, Saudi Arabia.
5. Department of Health Informatics, College of Public Health and Health Informatics. University of Ha'il, Ha'il, Saudi Arabia.
6. Department of Public Health, College of Public Health and Health Informatics. University of Ha'il, Ha'il, Saudi Arabia.
7. Independent Researcher, Zhenjiang 212013, China.

Table S1. The susceptibility of *E. coli* strains (n = 153) to antimicrobial agents.

Antimicrobial agent	Resistant		Intermediate		Susceptible	
	**No.**	**(%)**	**No.**	**(%)**	**No.**	**(%)**
**Gentamicin (GEN)**	45	29.4	4	2.61	104	68.0
**Amikacin (AMK)**	53	34.6	2	1.31	98	64.1
**Cefalexin (CFX)**	38	24.8	4	2.61	111	72.5
**Cefpodoxime (CEP)**	37	24.2	7	4.58	109	71.2
**Chloramphenicol (CHL)**	115	75.2	3	1.96	35	22.9
**Marbofloxacin (MAR)**	38	24.8	5	3.27	110	71.9
**Enrofloxacin (ENR)**	108	70.6	4	2.61	41	26.8
**Imipenem (IMI)**	47	30.7	2	1.31	104	68.0
**Polymyxin B (POL)**	80	52.3	3	1.96	70	45.8
**Tetracycline (TET)**	106	69.3	4	2.61	43	28.1
**Piperacillin (PIP)**	90	58.8	2	1.31	61	39.9
**Trimethoprim-sulfamethoxazole (SXT)**	125	81.7	5	3.27	23	15.0

Table S2. The drug resistance patterns of MDR *E. coli* strains.

Pattern code	DRP	Isolate number	MAR index	Pattern code	DRP	Isolate number	MAR index
**P1**	W, X, Y, Z	6	1.000	P6b	MAR, ENR, IMI, POL, Z	3	0.580
**P2**	X, Y, Z CEP, IMI	6	0.920	P7	AMK, CHL, ENR, Z	4	0.500
**P2a**	W, Y, Z, GEN, CFX	3	0.920	P7a	Y, Z	15	0.500
**P3**	W, Y, Z, AMK	5	0.830	P7b	ENR, IMI, POL, Z	3	0.500
**P4**	W, Y, AMK, TET, SXT	4	0.750	P8	X, CEP, CHL	3	0.420
**P4a**	X, CHL, IMI, POL, Z	3	0.750	P8a	AMK, GEN, ENR, PIP, SXT	3	0.420
**P5**	X, CEP, CHL, POL, TET, SXT	9	0.670	P8b	CHL, ENR, Z	11	0.420
**P5a**	Y, Z, AMK, GEN,	5	0.670	P8c	ENR, POL, Z	3	0.420
**P5b**	Y, Z, MAR, IMI,	8	0.670	P9	CHL, ENR, PIP, SXT	10	0.330
**P6**	Y, AMK, GEN, IMI, TET	2	0.580	P9a	CHL, ENR, TET, SXT	6	0.330
**P6a**	Y, MAR, IMI, TET, SXT	4	0.580	P10	CHL, ENR, SXT	3	0.250

DRP; Drug resistance pattern, **W**; CEP, MAR, IMI**, X**; AMK, GEN, CFX; **Y**, CHL, ENR, POL; **Z**, TET, PIP, SXT

Table S3. Phytochemical composition of *S. rebaudiana* using GC–MS.

Peak	RT	Compound name	MW	Chemical structure
1	5.73	Methyl salicylate	152.15	$C_8H_8O_3$
2	7.84	Eugenol	164.2	$C_{10}H_{12}O_2$
3	21.12	n-Hexadecanoic acid	256.42	$C_{16}H_{32}O_2$
4	21.51	Tetradecanoic acid	228.37	$C_{14}H_{28}O_2$
5	21.94	Hexadecanoic acid ethyl ester	284.46	$C_{18}H_{36}O_2$
6	26.81	Oleic acid	282.46	$C_{18}H_{34}O_2$
7	27.66	Octadecanoic acid	284.48	$C_{18}H_{36}O_2$
8	28.30	Phytol	296.53	$C_{20}H_{40}O$
9	34.534	i-Propyl 6,9,12-hexadecatrienoate	292.5	$C_{19}H_{32}O_2$
10	35.21	1-Heptatriacotanol	536.94	$C_{37}H_{76}O$
11	35.65	Imiprothrin	318.46	$C_{17}H_{22}N_2O_4$
12	35.92	Isosteviol	318.46	$C_{20}H_{30}O_3$
13	35.28	1-Heptatriacotanol	536.94	$C_{37}H_{76}O$
14	35.52	Imiprothrin	318.37	$C_{17}H_{22}N_2O_4$
15	37.95	trans-Geranylgeraniol	290.47	$C_{20}H_{34}O$
16	41.64	Campesterol	400.68	$C_{28}H_{48}O$

RT; retention time, **Mw**; Molecular weight

Table S4. Antimicrobial agents and zone interpretive chart based on CLSI guidelines (CLSI, 2017).

Antimicrobial group	Antimicrobial agent	Abbreviations	Concentration range (μg/ml)	Breakpoints		
				S	I	R
**Aminoglycosides**	Amikacin	AMK	30	≥17	15-16	≤14
	Gentamicin	GEN	10	≥15	13-14	≤12
**Cephalosporins**	Cefalexin	CFX	30	≥21	19-20	≤18
	Cefpodoxime	CFP	10	≥21	18-20	≤17
**Chloramphenicol**	Chloramphenicol	CHL	30	≥18	13-17	≤12
**Fluroquinolone**	Enrofloxacin	ENR	5	≥21	17-20	≤16
	Marbofloxacin	MAR	5	≥20	15-19	≤14
**Carbapenem**	Imipenem	IMI	10	≥23	20-22	≤19
**Polymixins**	Polymyxin B	POL	300	≥12	9-11	≤8
**Tetracycline**	Tetracycline	TET	30	≥15	12-14	≤11
**Sulfonamide**	Trimethoprim-Sulfamethoxazole	SXT	1.25/23.75	≥30	26-29	≤25
***β*-lactam**	Piperacillin	PIP	100	≥21	18-20	≤17

Table S5. Primers used for PCR amplification.

Target gene	Primer sequences	References
stx1	ACACTGGATGATCTCAGTGG<br>CTGAATCCCCCTCCATTATG	(Dipineto et al., 2006)
stx2	CCATGACAACGGACAGCAGTT<br>CCTGTCAACTGAGCAGCACTTTG	(Dipineto et al., 2006)
*eae*A	ATGCTTAGTGCTGGTTTAGG<br>GCCTTCATCATTTCGCTTTC	(Wang et al., 2002)
*hyl*A	AACAAGGATAAGCACTGTTCTGGCT<br>ACCATATAAGCGGTCATTCCCGTCA	(Birošová et al., 2004)
*hyl*D	CTCCGGTACGTGAAAAGGAC<br>GCCCTGATTACTGAAGCCTG	(Rodriguez-Siek et al., 2005)
afa	GCTGGGCAGCAAACTGATAACTCTC<br>CATCAAGCTGTTTGTTCGTCCGCCG	(Birošová et al., 2004)
*pap*A	GACGGCTGTACTGCAGGGTGTGGCG<br>ATATCCTTTCTGCAGGGATGCAATA	(Birošová et al., 2004)
estIb	TGTCTTTTTCACCTTTCGCTC<br>CGGTACAAGCAGGATTACAACAC	(Müller et al., 2007)
*elt*B	GAACAGGAGGTTTCTGCGTTAGGTG<br>CTTTCAATGGCTTTTTTTTGGGAGTC	(Müller et al., 2007)
*agg*R	ACGCAGAGTTGCCTGATAAAG<br>AATACAGAATCGTCAGCATCAGC	(Müller et al., 2007)
$bla_{\text{IMP-7}}$	AAGGCAGTATCTCCTCTCATTTTC<br>ACTCTATGTTCAGGTAGCCAAACC	(Gheorghe et al., 2014)
$bla_{\text{IMP-25}}$	GCAGTATTTCCTCACATTTCCATAG<br>TCACCCAAATTACCTAGACCGTAG	(Gheorghe et al., 2014)
bla_{TEM}	GCGGAACCCCTATTTG<br>ACCAATGCTTAATCAGTGAG	(Gheorghe et al., 2014)
bla_{SHV}	TTATCTCCCTGTTAGCCACC<br>GATTTGCTGATTTCGCTCGG	(Gheorghe et al., 2014)
$bla_{\text{OXA-2}}$	ATACACTTTTTGCACTTGATGCAG<br>TGAAAAGATCATCCATTCTGTTTG	(Gheorghe et al., 2014)
*tet*A	GGCGGTCTTCTTCATCATGC<br>CGGCAGGCAGAGCAAGTAGA	(Boerlin et al., 2005)
*aad*A	GTGGATGGCGGCCTGAAGCC<br>AATGCCCAGTCGGCAGCG	(Boerlin et al., 2005)
aac(3)-IV	AGTTGACCCAGGGCTGTCGC<br>GTGTGCTGCTGGTCCACAGC	(Maynard et al., 2004)

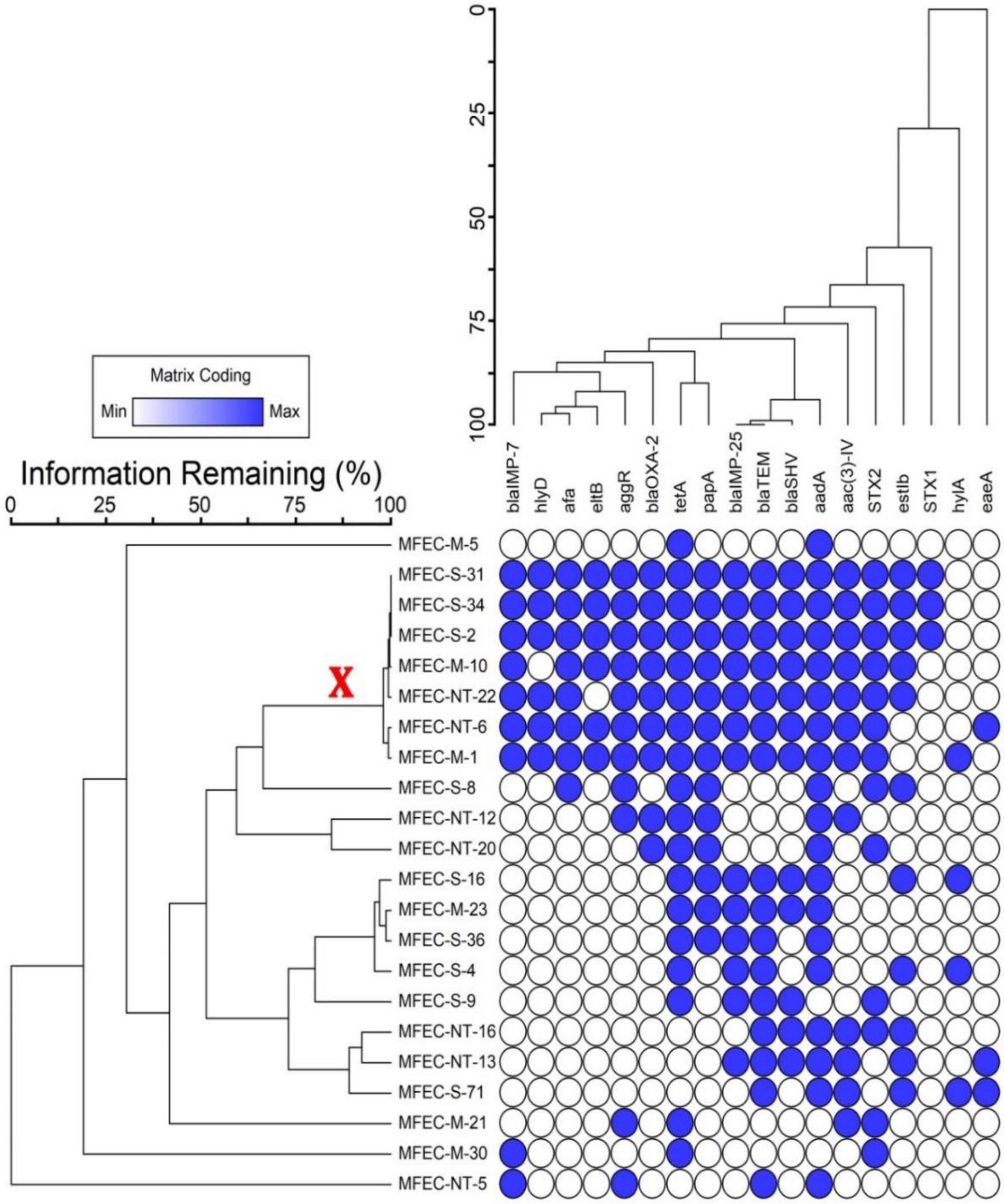

Figure S1. Clustering analysis Two-way dendrogram of pathogenic MDR *E. coli* strains using Sorensen methods for distance. ***afa***; afimbrial adhesion, ***elt*B**; heat-labile enterotoxin, ***estIb***; heat stable enterotoxin, $\boldsymbol{bla}_{\mathbf{IMP\text{-}7}}$, $\boldsymbol{bla}_{\mathbf{IMP\text{-}25}}$, $\boldsymbol{bla}_{\mathbf{TEM}}$ and $\boldsymbol{bla}_{\mathbf{SHV}}$; β-lactamase genes, $\boldsymbol{bla}_{\mathbf{OXA\text{-}2}}$; extended-spectrum β-lactamase, ***eae*A**; intimin, ***agg*R**; aggregative virulence regulator, ***hly*D**; cytolytic protein toxin, ***hyl*A**; enterohemolysin, ***pap*A**; P fimbria, ***stx***; Shiga toxin, and ***tet*A**; tetracycline resistance, ***aad*A** and ***aac*(3)-IV**; aminoglycosides resistance.

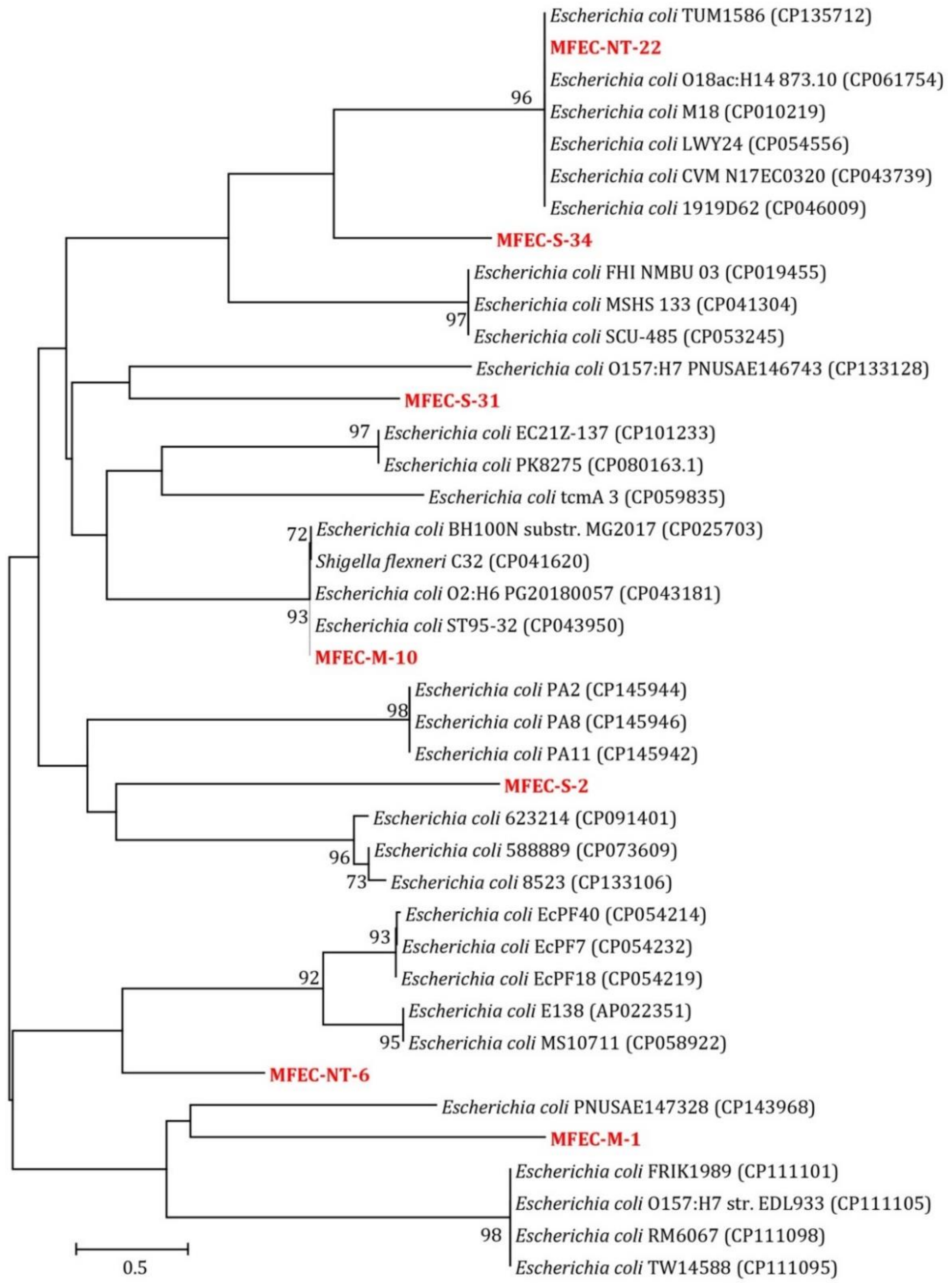

Figure S2. Neighbor-Joining tree constructed from 16S rRNA gene sequences, illustrating the phylogenetic placement of selected pathogenic MDR *E. coli* strains among closely related taxa.

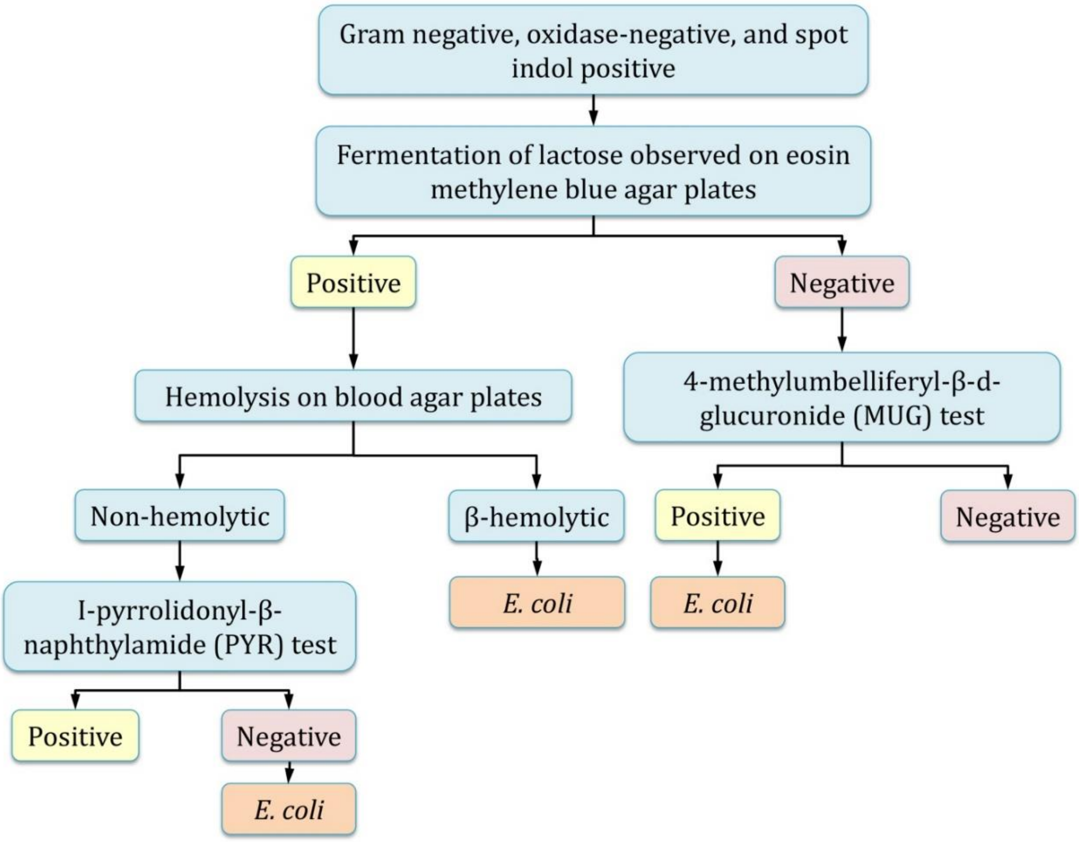

Figure S3. The scheme used for *E. coli* identification (York et al., 2000).

References

Birošová, E., Siegfried, L., Kmet'ová, M., Makara, A., Ostró, A., Grešová, A., Urdzík, P., Liptáková, A., Molokáčová, M., Bártl, R., Valanský, L., 2004. Detection of virulence factors in α-haemolytic Escherichia coli strains isolated from various clinical materials. Clin. Microbiol. Infect. 10, 569–573. https://doi.org/10.1111/j.1469-0691.2004.00922.x

Boerlin, P., Travis, R., Gyles, C.L., Reid-Smith, R., Janecko, N., Lim, H., Nicholson, V., McEwen, S.A., Friendship, R., Archambault, M., 2005. Antimicrobial resistance and virulence genes of Escherichia coli isolates from swine in Ontario. Appl. Environ. Microbiol. 71, 6753–6761. https://doi.org/10.1128/AEM.71.11.6753-6761.2005

Clinical and Laboratory Standards Institute standards (CLSI), 2017. Performance standards for antimicrobial susceptibility testing. CLSI Supplement M100 (27th edn), Clinical Laboratory Standards Institute, Wayne, PA

Dipineto, L., Santaniello, A., Fontanella, M., Lagos, K., Fioretti, A., Menna, L.F., 2006. Presence of Shiga toxin-producing Escherichia coli O157:H7 in living layer hens. Lett. Appl. Microbiol. 43, 293–295. https://doi.org/10.1111/j.1472-765x.2006.01954.x

Gheorghe, I., Czobor, I., Chifiriuc, M.C., Borcan, E., Ghiţă, C., Banu, O., Lazăr, V., Mihăescu, G., Mihăilescu, D.F., Zhiyong, Z., 2014. Molecular screening of carbapenemase-producing Gram-negative strains in Romanian intensive care units during a one year survey. J. Med. Microbiol. 63, 1303–1310. https://doi.org/10.1099/jmm.0.074039-0

Maynard, C., Bekal, S., Sanschagrin, F., Levesque, R.C., Brousseau, R., Masson, L., Larivière, S., Harel, J., 2004. Heterogeneity among virulence and antimicrobial resistance gene profiles of extraintestinal Escherichia coli isolates of animal and human origin. J. Clin. Microbiol. 42, 5444–5452. https://doi.org/10.1128/JCM.42.12.5444-5452.2004

Müller, D., Greune, L., Heusipp, G., Karch, H., Fruth, A., Tschäpe, H., Schmidt, M.A., 2007. Identification of unconventional intestinal pathogenic Escherichia coli isolates expressing intermediate virulence factor profiles by using a novel single-step multiplex PCR. Appl. Environ. Microbiol. 73, 3380–3390. https://doi.org/10.1128/AEM.02855-06

Rodriguez-Siek, K.E., Giddings, C.W., Doetkott, C., Johnson, T.J., Nolan, L.K., 2005. Characterizing the APEC pathotype. Vet. Res. 36, 241–256. https://doi.org/10.1051/vetres:2004057

Wang, G., Clark, C.G., Rodgers, F.G., 2002. Detection in Escherichia coli of the genes encoding the major virulence factors, the genes defining the O157:H7 serotype, and components of the type 2 Shiga toxin family by multiplex PCR. J. Clin. Microbiol. 40, 3613–3619. https://doi.org/10.1128/JCM.40.10.3613-3619.2002

York, M.K., Baron, E.J., Clarridge, J.E., Thomson, R.B., Weinstein, M.P., 2000. Multilaboratory validation of rapid spot tests for identification of Escherichia coli. J. Clin. Microbiol. 38, 3394–3398. https://doi.org/10.1128/JCM.38.9.3394-3398.2000
